# Drug Injection to Sites other than Arm: A Study of Iranian Heroin Injectors

**DOI:** 10.3389/fpsyt.2014.00023

**Published:** 2014-04-07

**Authors:** Mehrdad Karimi, Hafez Ghaheri, Shervin Assari, Khodabakhsh Ahmadi, Maryam Moghani Lankarani, Reza Moghani Lankarani, Hooman Narenjiha, Hassan Rafiey, Mahmood Tavakoli, Firoozeh Jafari

**Affiliations:** ^1^Shahrekord University of Medical Sciences, Tehran, Iran; ^2^Isfahan University of Medical Sciences, Isfahan, Iran; ^3^Department of Health Behavior and Health Education, University of Michigan School of Public Health, Ann Arbor, MI, USA; ^4^Behavioral Sciences Research Center, Baqiyatallah Medical Sciences University, Tehran, Iran; ^5^Universal Network for Health Information Dissemination and Exchange, Tehran, Iran; ^6^Medicine and Health Promotion Institute, Tehran, Iran; ^7^Substance Abuse and Dependence Research Center, University of Social Welfare and Rehabilitation Sciences, Tehran, Iran

**Keywords:** drug injection, injection sites, arm, Iran, heroin

## Abstract

For almost all injecting drug users (IDUs), the first site of injection is the arm. Years after injection, IDUs may shift to using other sites for intravenous (IV) access. Although injection to sites other than the arm is associated with higher risks, literature is limited regarding this behavior. We aimed to determine the prevalence and associated factors of using IV access points other than the arm among a national sample of IDUs in Iran. Data came from the National Drug Dependence Survey, 2007, which had enrolled 863 IDUs with at least one daily injection. Data on socio-demographics, pattern of drug use, and injection-related behaviors were entered into a logistic regression to determine predictors of injection to sites other than the arm. From all participants, 54.8% reported current injection sites in areas other than the arm. The other injection sites were the femoral venous sinus (17.0%), followed by the groin (14.5%) and neck (11.5%). Logistic regression revealed that living alone [odds ratio (OR) = 1.789, 95% confidence interval (CI) = 1.218–2.629], being Sunni (OR = 3.475, 95% CI = 1.775–6.801), having higher family income (OR = 1.002, 95% CI = 1.001–1.003), higher age at first drug use (OR = 1.039, 95% CI = 1.009–1.069), longer injection duration (OR = 1.071, 95% CI = 1.041–1.102), and more injection frequency (OR = 1.255, 95% CI = 1.072–1.471) were associated with higher likelihood of using injection sites other than the arm. Using sites other than the arm for IV injection is linked to socio-demographics, drug use data, and injection-related characteristics that can be used by policy makers. This information can be used for harm reduction planning.

## Introduction

Intravenous (IV) access points can develop several local complications that may involve skin or vascular structure (1–9). These complications vary in severity from a simple erythema and pain, to necrosis, thrombophlebitis, vein sclerosis, and occlusion. Following these complications, injecting drug users (IDUs) show interest in using other IV access points from an unusable vein to a new useable one (10, 11). Detailed information on IV access points increases our knowledge about IDUs’ high-risk behaviors. Clinicians, public health practitioners, and policy makers may use such information to protect the health of IDUs.

Although we know that first injections often begin in the arm as the primary point for IV access (10), there is lack of knowledge on trajectory of IDUs in using different IV access points. According to the current literature, most IDUs begin their injecting practice using the arm and then gradually shift to other sites including forearm, upper arm, hand, neck, feet, leg, and femoral vein. Some researchers regard IV access point as a measure of the severity of problems of IDUs (11).

The purpose of this study was to determine prevalence and associated factors of non-arm injection among Iranian IDUs using heroin.

## Materials and Methods

### Design and setting

Data came from the National Drug Dependence Survey that was conducted in 2007 and used a cross-sectional design. The main survey had a sample size of 7,743 performed by the research center for substance abuse and dependence at the University of Social Welfare and Rehabilitation Sciences. Some other manuscripts have been published from this database (12, 13).

### Participants and sampling

The participants were substance-dependent adults according to the DSM-IV. Participants were sampled from treatment centers (*n* = 1,217), prisons (*n* = 584), or streets (*n* = 5,860) from the capitals of 29 provinces in Iran. The sampling strategy in treatment centers and prisons was random, but snowball in streets. The number of samples taken from every province was proportional to the population of the province. The sampling started in April 2007 and lasted for 5 months.

### Process

The interviews were carried out by university graduates (MS, BS) with drug abuse-related majors/degrees who were dispatched to the provinces after being trained through workshops in Tehran (the capital of the Islamic Republic of Iran). Each interview took 60–90 min. Data were collected using a paper-based questionnaire that was the modified version of the one used in the previous national survey in Iran (14). The questionnaire included 69 items in nine sections.

### Secondary analysis

This study used a sample of all 863 IDUs with at least once daily heroin injections for 1 year. Participants were all heroin users with at least once daily heroin injections. We entered the following data into our analysis: socio-economic data, drug use pattern, and injection-related data.

### Outcome

“Injection to other sites than arm” was determined by the following question: To which site do you often inject? Responses included the femoral venous sinus, groin, neck, and other sites.

### Codes of ethics

The study was approved by the ethical review committee of the University of Social Welfare and Rehabilitation Sciences. Informed consent was obtained from all the participants after they had been verbally reassured that the information would be kept confidential, especially from the correctional system.

### Statistical analysis

Data were analyzed in the SPSS for Windows 13 statistical package. Continuous variables were described using mean and standard deviation (SD) or median (percentile 25% = Q1 and percentile 75% = Q3). In order to determine the association between independent variables and outcome (injection to sites other than the arm), the χ^2^ test, independent samples *t* test and Mann–Whitney were used. Logistic regression was used for multivariable analysis. Odds ratios (ORs), with 95% confidence intervals (CIs) were reported. *P*-value <0.05 was considered significant.

## Results

### Demographics and drug-related data

From the total 863 IDUs who were enrolled into this study, most participants were mostly men, Muslim, and had started drug use with opioids (Table 1).

**Table 1:** **Socio-demographic, drug related, and drug problems among IDUs (*n* = 863)**.

	*n*	%
**SAMPLING PLACE**		
Street	693	80.3
Prison	59	6.8
Treatment centers	101	11.7
Missed	10	1.2
**SAMPLING PROVINCE**		
Tehran	206	23.9
Khorasan	66	7.6
Guilan	63	7.3
Yazd	61	7.1
Lorestan	48	5.6
Hamedan	40	4.6
Kordestan	35	4.1
Fars	34	3.9
Hormozgan	32	3.7
Others	278	32.2
**SOCIO-DEMOGRAPHIC DATA**		
**Gender**
Men	835	96.8
Women	25	2.9
Missed	3	0.3
**Religion**
Islam	849	98.4
Shia	797	92.4
Sunni	52	6.0
**Other religions**	1	0.1
Missed		
Unemployed	371	43.0
Employed	0	0
Missed		
**Living alone**
With others	688	79.7
Alone	175	20.3
Missed	0	0
**DRUG USE DATA**		
**First drug of abuse**
Cannabis	289	33.5
Opium or its derivates	296	34.3
Heroin	97	11.2
Amphetamines	2	0.2
Others	173	
**Current dominant drug**
Powder heroin	498	57.7
Crystal heroin	365	42.3
**Years injection**
1–4	459	53.2
5–9	220	25.5
10–14	105	12.1
15–19	40	4.7
20 and more	30	3.1
**Monthly injection frequency**
3 times or less	24	2.8
4–30 times	64	7.4
31–60 times	104	12.1
61–90 times	428	49.6
90 times or more	243	28.2

Participants had a mean age of 31.7 ± 8.6 years. First drug use and injection had begun at 18.1 ± 5.3 and 25.7 ± 6.6, respectively. Injection duration was 6.0 ± 5.9 years. Mean income and drug payment were US $ 306.0 (835.3) and US $ 218.9 (300.5), respectively.

### Injection to other sites

Sites other than the arm were reported to be the site of injection among 54.8% of IDUs (*n* = 473). Injection sites included the groin (*n* = 125; 14.5%), neck (*n* = 99; 11.5%), femoral venous sinus (*n* = 147; 17.0%), and others (*n* = 26, 3.0%).

Based on our bivariate analysis, being Sunni (OR = 1.406, 95% CI = 1.187–1.667) and living alone (OR = 1.218, 95% CI = 1.057–1.403) were associated with higher likelihood of non-arm injection. Although men had a higher rate of non-arm injection than women, this difference was not significant (OR = 1.540, 95% CI = 0.910–2.607).

Logistic regression revealed that living alone (OR = 1.789, 95% CI = 1.218–2.629), being Sunni (OR = 3.475, 95% CI = 1.775–6.801), higher family income (OR = 1.002, 95% CI = 1.001–1.003), higher age at first use (OR = 1.039, 95% CI = 1.009–1.069), longer injection duration (OR = 1.071, 95% CI = 1.041–1.102), and more injection frequency (OR = 1.255, 95% CI = 1.072–1.471) were associated with an increased likelihood of injection to IV access points other than the arm (Table 2).

**Table 2:** **Regressors of non-arm injection among Iranian IDUs (*n* = 863)**.

Characteristics	*B*	SE	Wald	df	Sig.	Exp(*B*)	95% CI for EXP(*B*)	
							Lower	Upper
Living status (alone)	0.582	0.196	8.781	1	0.003	1.789	1.218	2.629
Islam type (Sunni)	1.245	0.343	13.210	1	0.000	3.475	1.775	6.801
Family income ($ US)	0.001	0.000	7.367	1	0.007	1.001	1.000	1.001
Age at first use (years)	0.038	0.015	6.572	1	0.010	1.039	1.009	1.069
Injection duration (years)	0.069	0.014	22.713	1	<0.001	1.071	1.041	1.102
Past year injection frequency	0.227	0.081	7.939	1	0.005	1.255	1.072	1.471

## Discussion

More than half of Iranian male IDUs use IV access points other than the arm. Logistic regression revealed that likelihood of other IV access points other than the arm was increased by some socio-economic characteristics such as living alone (OR = 1.789), being Sunni (OR = 3.475), and higher family income ($ US) (OR = 1.002). Drug-related data such as higher age at first drug use (OR = 1.039), and injection-related data such as more injection duration (OR = 1.071) and injection frequency (OR = 1.255) were also linked to this behavior.

From the above list of factors, the effect of injection duration and injection frequency on likelihood of shifting to other sites of injection was expected, as shift in IV access point occurs as drug injection progresses. That is, the shift in site of drug use may be a part of the natural history of drug injection. However, in the very limited pool of published evidences about pattern of IV access points, we could not find any evidence confirming or rejecting our findings about the association of being Sunni, family income, age at first drug use, or our outcome.

In contrast to our expectations, and opposite to the literature that emphasizes the role of low socio-economic status as a risk factor of poor health (15–18) and health risk behaviors (19, 20), our study suggested that IDUs with higher family income may be at higher risk of shifting to using other IV access points.

Different from most of the literature, it was not lower age at first drug use (21, 22) but higher age at first drug use that was associated with higher risk of shifting from the arm to another injection site. Similar to our findings, one study on 144 IDUs with ≤5 years of injection use reported that adolescent-initiating IDUs were less likely to report high-risk sex and injection behaviors than adult-initiating IDUs (23). Baldwin et al. (24) reviewed the literature and hypothesized that early initiation of substance use may be associated with a pattern of maladaptive coping mechanisms in which the substance is implemented to manage stress (25). Heavy drug use, during a critical period of neurobiological development, may also lead to dysfunction in memory and learning, inhibition, and executive functioning and neuronal activation, or even ultimately alterations in brain structure (26).

The use of injection sites other than the arm among IDUs is a major public health concern. Such behaviors are often associated with an increased risk of vascular complications such as deep vein thrombosis, leg ulcers, and vascular insufficiency. In addition, in some sites such as the groin and neck, the close proximity to other organs poses the risk of inadvertent trauma to these sites (27). Unfortunately, data is systematically scarce in this regard (10).

Some years after first injection (10), IDUs may have some concerns about difficulty of injection in previous injection sites, and worry about the risk of loss of the injection/hit. In this stage, other sites might be seen by an IDU as “easy-to-use” with more secure delivering of the injection intravenously (10). An IDU may show interest in using other points for IV access, as a result (11).

Within non-arm injection sites, the most frequent site was the groin. According to the literature, among heroin IDUs, groin injection seems convenient, providing quick access, with little mess and less pain than smaller more awkward veins. The formation of sinuses over time facilitates continued use of the groin (10).

According to our findings, likelihood of non-arm injection increases by an increase in injection duration. Literature confirms that most IDUs begin to use other injection sites years after their first injection (10). According to a study, IDUs shift to injection in the forearm in 2 years, shift to injection in the upper arm in 3.5 years, shift to injection in the hand, neck, feet, and leg in 4 years, and shift to injection in the groin 10 years after their first injection (11). Injectors decide to shift to a new injection site when they believe the previous site is no longer accessible (10).

Based on harm reduction protocols, practices such as the rotation of injecting sites may prevent scar tissue occlusion, swelling, infections, and deep vein thrombosis among IDUs. In addition, literature cites that IDUs believe that this rotation is both difficult and unreliable. The rotation of injecting to both arms needs using non-dominant hands, which might seem problematic to IDUs. Secondary to this perceived difficulty, IDUs find other IV access points as convenient, providing quick access, with little mess and less pain than previously used arm veins (10).

Non-arm injection is of interest because it is linked to high rates of local complications including tissue damage, infections, and deep venous thrombosis (10, 11). Our recommendation to service providers is that site of injection is an important factor that should be asked about when a service is being delivered to an IDU. Harm reduction services in Iran should include safe injecting trainings for IDUs.

This study sheds more light on the risk factors of transition to more severe injection sites. Of the factors, injection frequency and living status are modifiable, and can be considered as the target of prevention programs. Injecting to sites such as the femur or neck should be strongly discouraged, because of the associated health hazards. These veins are at proximity of vital arteries. Missing the aimed vein may result in hitting the artery or nerves, which may cause major health problems for the IDU (28, 29). Promotion of safe injection should be considered as a part of harm education programs, and may encourage the practice of safe injection among IDUs. Such training programs can be delivered as a part of needle exchange programs.

Safe injecting education programs may benefit from information about the motivations for transitioning to new IV access points. We do not know whether training programs that enhance safe injection information among IDUs will increase the use of peripheral veins and decrease the use of risky injection sites such as neck and groin. IDUs should not be encouraged to use others for injection or injection facilitation, as presence of other IDUs at the time of injection may increase the risk of shared injection, which is linked to risk of blood-borne infections such as HIV. However, IDUs should receive information on health risks associated with transition to other access points for injections.

Not only may the findings of this research be of interest to the policy makers in Iran, but they may also benefit the global harm reduction community. Groin injecting is a neglected topic. Similar studies will shed more light on factors that are linked to similar practices among IDUs. Information provided by this study is hoped to be used for the development of evidence-based safe injecting advice.

## Limitation

Although Iran is a country that includes multiple ethnicities with unique cultures, we did not enter ethnicity to our analysis. Some participants were sampled using a snowball strategy, thus the results are not generalizable to all Iranian male IDUs. This study did not enquire data on the pattern of transition between different injection sites. The study only included heroin users, thus further research is needed on individuals who inject drugs other than heroin. This study had a retrospective design. Longitudinal studies are still required. Further work is needed to better understand the natural history of IDUs, especially their pattern of shift in IV access points.

## Conclusion

This study found that half of Iranian male IDUs report injection to IV accesses points other than the arm. This behavior is attached to some socio-economic and drug-related characteristics. Information on factors associated with non-arm injection may be used for evidence-based harm reduction practice.

## Conflict of Interest Statement

The authors declare that the research was conducted in the absence of any commercial or financial relationships that could be construed as a potential conflict of interest.
